# Calculation of standard bodyweights for dogs, cats, rabbits, and guinea pigs

**DOI:** 10.1371/journal.pone.0318734

**Published:** 2025-02-13

**Authors:** Stuart D. Becker, Dan G. O’Neill, Siân-Marie Frosini, Laura E. Stapleton, David M. Hughes, Dave C. Brodbelt

**Affiliations:** 1 Pathobiology and Population Sciences, The Royal Veterinary College, Hertfordshire, United Kingdom; 2 Vets for Pets UK Limited, Handforth, Cheshire, United Kingdom; 3 Department of Health Data Science, Institute of Population Health, University of Liverpool, Liverpool, United Kingdom; Ain Shams University Faculty of Agriculture, EGYPT

## Abstract

Standard bodyweights are an essential component of calculations that summarise many population-level measures in companion animals, including the defined daily doses for veterinary species (DDDVet) reporting antimicrobial usage. Standard species bodyweights may originate from data derived from clinical records, but current methods to obtain these values risk inaccuracy because they exclude measurements obtained from juvenile animals and consider only individuals that have achieved stable adult bodyweight. This study aimed to improve the accuracy of standard population level species bodyweights through the development of a prediction modelling approach to estimate point mean population bodyweight in dogs, cats, rabbits, and guinea pigs. Data were obtained from the VetCompass database and included bodyweight measurements from approximately three million dogs, two million cats, 220,000 rabbits and 62,000 guinea pigs across 1,800 veterinary practices in the United Kingdom. Initially, Loess models were used to identify the age at which juvenile animals transitioned from growth to stable adult bodyweight. Linear mixed effects models were developed to predict juvenile growth, calibrated such that predicted cessation of growth matched that observed in the Loess models. The prediction models were then used to adjust bodyweight measurements obtained from clinical records of juvenile patients, allowing historical measurements to be included for estimation of a point mean population bodyweight on a subsequent specified target date. Juvenile growth transitioned to stable adult bodyweight at approximately 14 months in dogs, and 13 months in cats, rabbits, and guinea pigs. Point mean whole-population bodyweights estimated on 31^st^ December for each year 2014 – 2023 found that the mean bodyweight of cats, rabbits, and guinea pigs was approximately 4.2 kg, 2.3 kg, and 1.0 kg respectively and changed little over this time period. However, dogs showed a trend to lower mean bodyweight over time, with a mean value of 17.6 kg in 2014, reducing to 16.1 kg by 2023.

## Introduction

Standard bodyweights have a range of uses in companion animal species, including benchmarking and evaluation of drug utilisation [1], epidemiological monitoring of health trends in animal populations [2], development of nutritional guidelines [3], and contribution to determining insurance premiums [4]. Mean species bodyweights may be derived from electronic health records, but current methods are unlikely to reflect the mean bodyweight of the whole population because they rely on adult patients only and do not include measurements obtained from juvenile animals prior to achieving stable adult bodyweight [1].

Standard bodyweights are essential for population-level monitoring of medication usage in both human and animal populations as part of the ‘One Health’ framework [5]. This type of surveillance is recognised by the World Health Organisation as a vital tool to derive “comparable drug utilisation statistics” [6]. In people, standard measures of drug utilisation have been used to evaluate and compare national drug policies, prescribing patterns, procurement logistics, and adverse drug reactions, across different populations and time periods [6]. In veterinary patients, national surveillance of antimicrobial use in particular is critically important to understanding and mitigating the risk of antimicrobial resistance [1], and collection of sales data is a statutory requirement in the United Kingdom [7] and European Union [8]. A reliable and comparable measure of drug utilisation is essential to allow accurate monitoring of trends in drug use across different populations and time periods.

A commonly-used measure to monitor medication usage is the ‘Defined Daily Dose’ (DDD), which for human patients is defined as “the assumed average maintenance dose per day for a drug used for its main indication in adults” [9]. For anti-infectives, annual usage at a population level is commonly expressed as DDD per individual at risk per year, which estimates the number of days per year for which each individual in a population receives treatment on average.

In order to calculate the DDD in human patients, WHO guidelines assume a standard adult bodyweight of 70 kg [9]. While the WHO has recognised that dose recommendations for children vary with age and bodyweight, the difficulty in defining such doses for paediatric patients means that adult DDD values are used for all human patients regardless of age under most circumstances [9]. As the rate of prescribing for many drugs is similar in juveniles and adults [10] and there is substantial variation in age distribution between national populations around the world [11], ignoring bodyweight differences between juveniles and adults could make comparison of drug use between different populations challenging.

Similar challenges occur when estimating standard bodyweights in companion animal populations. A substantial proportion of companion animal patients are juveniles that have not yet reached their full adult bodyweight [12]. Additionally, some companion animal species exhibit vast phenotypic diversity due to historic selective breeding practices, and so different breeds may exhibit a wide range of bodyweights [13]. Sources of data on companion animal population bodyweights are limited, but in the UK suitable data can be extracted from two large databases derived from veterinary electronic health records: SAVSNET [14] and VetCompass [15]. Typical adult breed bodyweight ranges for dogs are also described in Kennel Club breed standards [16], although cross-breeds are not included in this source, and juvenile bodyweights are not described.

Inclusion of juvenile animals in mean species bodyweight calculations would ensure that all patient age groups are represented. This is feasible because clinical records are date-stamped, allowing bodyweight data to be combined with the dates of birth of each animal and aggregated across large numbers of individuals to calculate mean population bodyweights. However, selection of the most appropriate bodyweights from electronic health records to include in this calculation is not straightforward.

Veterinary patient bodyweights are recorded on an ad-hoc basis when animals are weighed by veterinary practices. As bodyweight may be recorded on multiple occasions during each patient’s lifetime under veterinary care, clinical records often contain bodyweight measurements on multiple dates for each individual animal. Each animal may contribute only one measurement to mean species bodyweight, and so a single bodyweight must be obtained for each individual (e.g., by selecting one recorded measurement and excluding others, or by taking an average of measurements for each individual). As these bodyweight values are collected from different individuals at different times, calculating a mean population bodyweight by averaging individual bodyweights obtained from clinical records results in a ‘period mean’ (taken over a period of time), and not a ‘point mean’ (relating to a specific point in time).

The period mean and point mean values are unlikely to be equivalent due to the growth of juvenile animals over time. As bodyweights generally rise and then plateau over a lifetime, a population’s period mean that includes historical bodyweight records from juvenile animals is likely to be lower than the point mean reflecting true bodyweight values on a subsequent specific date, due to bodyweight increase associated with juvenile growth. Further, the frequency of bodyweight measurement may vary between veterinary practices and patient age groups, resulting in variation in age distribution that reduces comparability between subpopulations of animals. Thus, point mean population bodyweight that represents the population status at a specific point in time, is preferred for use in calculations such as the defined daily dose for veterinary species (termed the DDDVet) [1,17,18].

To approximate a point mean population bodyweight, previous reports of antimicrobial use in dogs and cats have followed the methods used to calculated DDD in humans [6], and calculated the DDDVet per animal using only adult bodyweights extracted from the SAVSNET database [14], excluding patients under two years of age [1,17]. While this straightforward approach avoids concerns about changes in juvenile patient bodyweight over time, selective inclusion only of animals that have achieved full adult bodyweight is likely to systematically overestimate the point mean bodyweight of the population overall and bias estimates of drug utilisation per animal downwards (S1 Formula). Conversely, calculation of a period mean bodyweight using records from patients of all ages is likely to underestimate the point mean bodyweight for the population overall as historical recorded bodyweights do not account for subsequent juvenile growth.

Here, we describe a new approach to generate representative, population-level standard annual species bodyweights suitable for use in calculations of DDDVet per animal [1]. To facilitate future DDDVet calculations, we propose to disseminate standard point mean full population species bodyweights via the Royal Veterinary College VetCompass website [15] updated on an annual basis.

## Methods

### Data extraction and population

Data were extracted from the VetCompass database [15] using Microsoft SQL Server Management Studio (Version 19.3) [19], comprising bodyweight measurements recorded during invoiced episodes of care identified by a unique invoice number between the years 2014 and 2023. Restricting measurements to those associated with an invoiced episode of care aimed to reduce the risk that bodyweights guessed by practice staff when the patient was not present would be included in the data set. Information was extracted on each patient with at least one recorded bodyweight, and included an individual animal identification code, species, sex, birth date, and the dates and recorded values of all bodyweight measurements. Identification codes for veterinary practices and groups were recorded to assess consistency of mean population bodyweight estimates between different organisations. Ethics approval was obtained from the RVC Social Science Research Ethical Review Board (reference number SR2022-0095). A pilot survey identified that the most commonly examined species in the VetCompass database from Jan 1^st^ 2021 to Dec 31^st^ 2023 were dogs (59.5%), cats (35.8%), rabbits (2.6%), and guinea pigs (0.8%).

Animals were excluded from the analytic data sets used for population bodyweight calculations if their date of birth or at least one bodyweight was not recorded, if their recorded bodyweight or age was deemed unrealistic for their species (Table 1), or if the recorded monthly bodyweight increase was unrealistically high (over ten times the previous month’s bodyweight for dogs and cats, or over three times the previous month’s bodyweight for rabbits and guinea pigs) [20–23].

**Table 1:** Bodyweight and age ranges considered realistic for inclusion in data set used to estimate full-population point mean species bodyweights.

Species	Bodyweight range (kg)	Age range (years)
**Dog**	0.07–125 [1,20]	0–22.5 [17]
**Cat**	0.07–22 [1,21]	0–27.5 [17]
**Rabbit**	0.035–25 [24,25]	0–19.0 [26]
**Guinea pig**	0.04–1.4 [27,28]	0–15.0 [29]

The data used to develop statistical models included all bodyweight measurements associated with an invoice in 2022–2023 (dogs) or 2021–2023 (cats, rabbits, guinea pigs). These time periods were selected to maximise sample size while avoiding developing models using information collected during the Covid-19 pandemic, where opportunities for bodyweight measurement may have been abnormally infrequent in many practices [30].

A similar data set was extracted for validation, that included patients weighed during the period 2018–2020. To calculate annual point mean population bodyweight estimates over time, bodyweight measurements were also extracted for all dogs, cats, rabbits, and guinea pigs with invoiced episodes of care separately for each year between 2014 and 2023.

All statistical analyses were undertaken using R (Version 4.3.3) [31], with plots generated using the ‘ggplot2’ package [32].

### Summary of analytical approach

Estimation of point mean population bodyweight that accounts for juvenile growth was achieved in four stages. Initially Loess models [33] were used to define from observed data the age at which the juvenile growth period transitioned into stable adult weight for each species. Secondly, linear mixed effects models were developed to predict the magnitude and pattern of change in bodyweight expected during the identified juvenile growth phase. Thirdly, the proportional increase in bodyweight was estimated for all individual juvenile animals from their last recorded weight in the clinical record prior to the target date for point mean calculation. For adult animals, the most recent recorded bodyweight prior to the point mean target date was included without adjustment. Finally, the point mean species bodyweight was calculated from the estimated point bodyweights of all individual animals at the target date of 31^st^ December for each year for annual reporting.

### Estimation of duration of juvenile growth period

The age range for the period of juvenile growth from birth to the end of puberty [34] in dogs, cats, rabbits, and guinea pigs was estimated using Loess models [33] within the fANCOVA package [35], that generated patient bodyweight predicted by age in months with the smoothing parameter selected by Akaike information criterion (AIC) or generalised cross-validation (GCV).

Loess models were used to predict the increase in patient bodyweight over time for juvenile dogs, cats, rabbits, and guinea pigs, with the end of juvenile growth interpreted as the earliest age at which the predicted interval change in bodyweight was zero at an interval resolution of 0.05 months.

### Estimation of magnitude and pattern of proportional change in juvenile bodyweight over time

**Fig 1:**
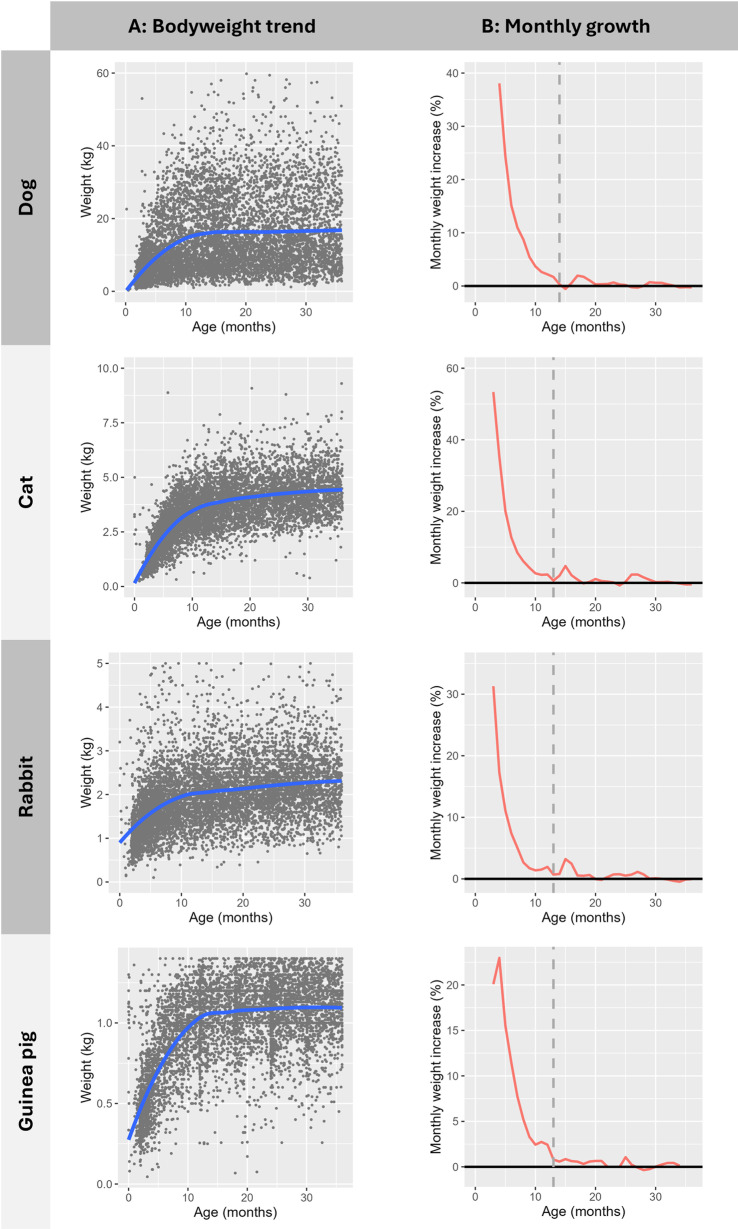
Juvenile growth by species. Loess smoother with standard parameters [32] (A: bodyweight trend) and optimised parameters (B: monthly growth). Dashed line indicates end of main juvenile growth phase, precise age not visible on all plots at monthly resolution. Scatter plots include a random sample of 10,000 animals per species.

Linear mixed effects regression was used to model the change in bodyweight of juvenile animals of each species over time. To account for wide variation in typical absolute bodyweight values between dog breeds, the outcome variable was proportional bodyweight gain from the previous measurement for each animal. It was anticipated that the rate of growth would be lower in older compared to younger patients, and that a longer interval between bodyweight measurements would be associated with greater change in bodyweight. Therefore, predictors for model development included age at bodyweight measurement (months) and interval between the date of bodyweight measurement and target date for point mean calculation (months), with random effects to address potential correlation between multiple observations in individual animals. As initial examination of the data showed a non-linear relationship between bodyweight and age, with more rapid growth in younger animals (Fig 1), the effects on model fit of including quadratic and cubic polynomial terms and an interaction term were investigated for both age and interval (S2 Formula). Backward selection was used to generate the final parsimonious models retaining predictors based on AIC via the MASS package [36].

Model fit was assessed by calibration-in-the-large, calibration slope and visual examination of calibration plots, and internal validation was performed by bootstrapping using 100,000 repetitions and 1% of patients per repetition with replacement for each species model. While some individual patients may be included in the development (years 2021–2023) and validation data sets (years 2018-2020), most animals classified as juvenile in 2018–2020 would have achieved full adult bodyweight by 2021–2023, and thus few individual animals would have been included in the juvenile phase in both data sets. Calibration of models using validation data sets was assessed as for development data sets.

Sensitivity testing of the linear regression models was undertaken by varying the age range of animals included in the model development data sets. This demonstrated that the predicted maximum age for juvenile growth increased when older animals were included in the development data set. Thus, results from the Loess models were used to guide the age range of animals included in the data set for linear regression model development. To calculate the optimal age range for each development data set, multiple segments of the development data set were created where the minimum patient age was set as zero and the maximum integer age value in months obtained using a binary search algorithm [37]. For each iteration updated coefficients were substituted into the polynomial equation describing the model, which was then solved for age assuming an interval of one month. Equations were solved using Wolfram Alpha [38] which allowed rapid processing of large numbers of complex formulae. After optimisation of the development data set age range, the end of juvenile growth for each species predicted by the linear mixed effects models closely matched those found in the Loess models that were derived from observed data.

### Calculation of estimated annual point mean population bodyweights

Point mean population bodyweights were calculated for each calendar year 2014–2023 for dogs, cats, rabbits, and guinea pigs, using a target date of 31^st^ December for each reported year. Targeting of the last day of each year aimed to achieve the most representative annual mean bodyweight by maximising the number of bodyweight measurements in each annual data set. For juvenile animals, the most recently recorded bodyweight prior to 31^st^ December was adjusted by predicting the proportional change in bodyweight between the measurement date and the target date for point mean calculation, or the end of juvenile growth, whichever occurred first. For adult animals the most recently recorded bodyweight prior to 31^st^ December was used without adjustment. Point mean species bodyweights were compared between different practices and contributing veterinary groups using Kruskal-Wallis tests.

To investigate the potential effect of bias on estimates of mean species bodyweights in previous studies reporting antimicrobial use in small companion species, which excluded juvenile animals when calculating DDDVet per animal [1,17], bodyweights for each annual data set were also calculated for a sub-population of animals over two years of age. Additionally, period mean bodyweight was calculated for all animals without adjustment for juvenile growth, to assess the difference between period and point mean bodyweight estimation calculated from the same underlying data.

## Results

### Data sets

Data extracted for development of the Loess models to estimate age at the end of juvenile growth included bodyweight measurements from 2,908,075 dogs, 1,996,695 cats, 219,416 rabbits and 62,219 guinea pigs (Table 2). Data were available from between 1,292 and 1,798 unique veterinary practices depending on the range of species seen by each practice, spread across seven large veterinary groups. Both age and bodyweight distributions were skewed to the right for all species, and there were similar numbers of males and females within all four species (percent female: dog 47.5%, cat 50.0%, rabbit 45.0%, guinea pig 46.3%).

**Table 2:** Size and characteristics of data sets used to develop Loess models to determine age at end of juvenile growth, and to develop and validate regression models predicting pattern of juvenile growth over time.

Data set	Data period	Species	Unique patients	Unique bodyweight measurements	Unique practices	Bodyweight (kg) median (interquartile range)
**Estimation of duration of juvenile growth period**	1 Jan 2022 –31 Dec 2023	Dog	2,908,075	25,523,484	1,602	12.0 (7.1, 22.1)
	1 Jan 2021 –31 Dec 2023	Cat	1,996,695	13,922,115	1,798	3.9 (3.0, 4.8)
		Rabbit	219,416	1,124,002	1,576	2.0 (1.5, 2.5)
		Guinea pig	62,219	162,860	1,292	1.0 (0.8, 1.1)
**Estimation of magnitude and pattern of proportional change in juvenile bodyweight over time** **(model development)**	1 Jan 2022 –31 Dec 2023	Dog	1,259,653	6,831,397	1,513	11.3 (6.5, 20.4)
	1 Jan 2021 –31 Dec 2023	Cat	829,496	3,663,494	1,548	3.6 (2.5, 4.5)
		Rabbit	101,247	392,268	1,326	2.0 (1.5, 2.5)
		Guinea pig	23,791	58,707	1,201	1.0 (0.8, 1.2)
**Estimation of magnitude and pattern of proportional change in juvenile bodyweight over time** **(model validation)**	1 Jan 2018 –31 Dec 2020	Dog	1,627,995	9,869,857	507	11.0 (6.4, 21.2)
		Cat	908,569	4,047,699	506	3.9 (3.0, 4.7)
		Rabbit	227,063	776,985	1,532	2.0 (1.5, 2.5)
		Guinea pig	57,048	120,861	1131	1.0 (0.7, 1.1)

### Age at end of juvenile growth

In all species examined the juvenile phase was associated with early-life rapid growth, which slowed over time and stabilised to mature adult bodyweight (Fig 1A). Optimisation of the Loess smoothing factor demonstrated that the juvenile growth phase ended at 13.80 months of age in dogs, 12.80 months in cats, 13.00 months in rabbits, and 12.80 months in guinea pigs (Fig 1B).

### Magnitude and pattern of proportional juvenile growth over time

Following backward selection, all predictors (age, interval, and their interaction and polynomial terms) were retained for cats, rabbits, and guinea pigs, while in dogs the cubic polynomial term for the interval between bodyweight measurement and point mean bodyweight target date was not significant and so was excluded from the final model for this species (Table 3). To match the end of juvenile growth identified from the Loess models, the data sets used to develop the linear mixed effects regression models included animals up to 59 months of age in dogs, 58 months in cats, 50 months in rabbits, and 57 months in guinea pigs (Table 2). The end of juvenile growth predicted by the linear mixed effects models closely approximated values obtained from the Loess models (dog 14.01 months, cat 12.98 months, rabbit 13.07 months, guinea pig 13.01 months). Internal and external model calibration was good in all cases (calibration-in-the-large intercept ≈  0 in all cases; calibration slope (95% confidence interval): dogs 0.99 (0.989, 0.993), cats 1.03 (1.031, 1.037), rabbits, 0.99 (0.983, 0.998), guinea pigs 1.03 (1.007, 1.059) based on validation data set for more conservative estimate). Bootstrapping found negligible model optimism. Variation in proportional growth over time between patients within each species group was negligible (variance partition coefficient for individual patients was less than 10^−7^ for all species).

**Table 3:** Coefficients of linear mixed effects models predicting proportional bodyweight gain in the context of a known starting age and bodyweight, and time interval for growth.

Predictor	Coefficient (95% confidence interval)			
	**Dog**	**Cat**	**Rabbit**	**Guinea Pig**
Intercept	0.385 (0.385, 0.386)	0.376 (0.375, 0.377)	0.183 (0.180, 0.186)	0.109 (0.104, 0.114)
Age	−0.053 (−0.053, −0.052)	−0.055 (−0.056, −0.055)	−0.031 (−0.031, −0.030)	−0.018 (−0.018, −0.017)
Age^2^	0.002 (0.002, 0.002)	0.002 (0.002, 0.002)	0.001 (0.001, 0.001)	0.001 (0.001, 0.001)
Age^3^	−0.00002 (−0.00002, −0.00002)	−0.00002 (−0.00002, −0.00002)	−0.00001 (−0.00002, −0.00001)	−0.00001 (−0.00001, −0.00001)
Interval	0.065 (0.065, 0.065)	0.072 (0.072, 0.072)	0.056 (0.055, 0.057)	0.041 (0.039, 0.043)
Interval^2^	−0.001 (−0.001, −0.001)	−0.002 (−0.002, −0.002)	−0.002 (−0.002, −0.002)	−0.001 (−0.002, −0.001)
Interval^3^	–	0.00004 (0.00004, 0.00004)	0.00003 (0.00002, 0.00003)	0.00002 (0.00002, 0.00003)
Age x interval	−0.002 (−0.002, −0.002)	−0.001 (−0.001, −0.001)	−0.001 (−0.001, −0.001)	−0.001 (−0.001, −0.001)

Age and Interval in months valid up to maximum juvenile age (dogs 14 months, other species 13 months). p <  0.0001 in all cases. Form of model is provided in S2 Formula.

### Point mean annual population bodyweights

**Fig 2:**
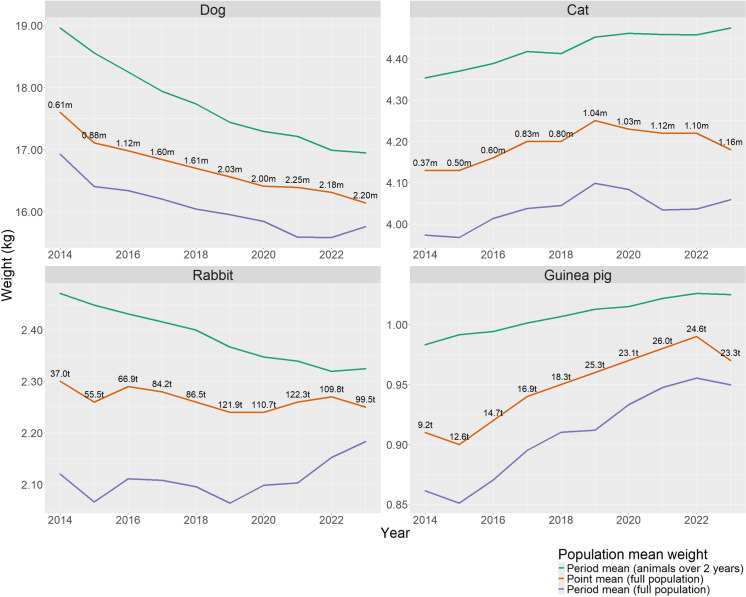
Estimates of population mean bodyweight by year and species obtained using different methods. ‘Period mean (animals over 2 years)’ bodyweights include recorded bodyweights from clinical records for animals over two years of age only. ‘Point mean’ bodyweights are calculated using recorded bodyweights in the clinical records for all animals adjusted for juvenile growth. ‘Period mean (full population)’ bodyweights are calculated from recorded bodyweights in the clinical records for all animals without adjustment. The number of animals included for calculation of each annual mean bodyweight value are shown in millions (m) or thousands (t).

Estimated annual point mean population bodyweights were calculated for dogs, cats, rabbits, and guinea pigs for each year between 2014 and 2023 (Fig 2; Table 4). The point mean population bodyweight of cats and rabbits remained relatively stable between 2014 and 2023 at around 4.2 kg and 2.25 kg respectively. The annual point mean population bodyweight of guinea pigs showed a small increase over time from 0.91 kg in 2014 to 0.97 kg by 2023. The annual point mean population bodyweight of dogs showed a downward trend from 17.6 kg in 2014 to around 16.1 kg by 2023. No significant differences in mean population bodyweight were detected for any species between different practices or veterinary groups (p =  NS in all cases).

**Table 4:** Annual point mean populations bodyweights (kg) calculated for target date of 31st December of each year using recorded bodyweights in the clinical records for all animals measured in the specified year, adjusted for juvenile growth.

Species	Year	Practices (n)	All patients (n)	Juveniles n (%)	Point mean animal bodyweight (kg) (95% confidence interval)
**Dog**	2014	837	610,881	107,482 (17.59)	17.60 (17.60, 17.60)
	2015	1,026	877,191	184,447 (21.03)	17.11 (17.11, 17.12)
	2016	1,286	1,120,196	216,388 (19.32)	16.98 (16.98, 16.98)
	2017	1,605	1,600,486	287,571 (17.97)	16.84 (16.84, 16.84)
	2018	1,741	1,613,142	311,975 (19.34)	16.70 (16.70, 16.70)
	2019	1,773	2,028,493	397,778 (19.61)	16.56 (16.56, 16.56)
	2020	1,910	1,996,720	378,200 (18.94)	16.41 (16.40, 16.41)
	2021	1,806	2,247,545	538,821 (23.97)	16.39 (16.39, 16.39)
	2022	1,580	2,177,312	525,973 (24.16)	16.31 (16.31, 16.32)
	2023	1,576	2,198,210	448,262 (20.39)	16.14 (16.14, 16.14)
**Cat**	2014	777	370,696	63,750 (17.20)	4.13 (4.13, 4.13)
	2015	954	502,219	98,900 (19.69)	4.13 (4.13, 4.13)
	2016	1,199	603,654	110,895 (18.37)	4.16 (4.16, 4.16)
	2017	1,508	833,665	149,056 (17.88)	4.20 (4.20, 4.20)
	2018	1,648	795,226	148,118 (18.63)	4.20 (4.20, 4.20)
	2019	1,694	1,041,353	195,463 (18.77)	4.25 (4.25, 4.25)
	2020	1,746	1,033,466	202,278 (19.57)	4.23 (4.23, 4.23)
	2021	1,705	1,117,280	262,135 (23.46)	4.22 (4.22, 4.22)
	2022	1,504	1,101,013	269,453 (24.47)	4.22 (4.22, 4.22)
	2023	1,496	1,159,288	278,571 (24.03)	4.18 (4.18, 4.18)
**Rabbit**	2014	590	36,994	15,013 (40.58)	2.30 (2.29, 2.30)
	2015	763	55,530	26,010 (46.84)	2.26 (2.26, 2.27)
	2016	1,016	66,941	28,424 (42.46)	2.29 (2.29, 2.29)
	2017	1,290	84,204	32,914 (39.09)	2.28 (2.27, 2.28)
	2018	1,414	86,518	34,828 (40.26)	2.26 (2.26, 2.26)
	2019	1,478	121,855	52,262 (42.89)	2.24 (2.24, 2.24)
	2020	1,449	110,673	41,275 (37.29)	2.24 (2.24, 2.24)
	2021	1,445	122,262	46,741 (38.23)	2.26 (2.25, 2.26)
	2022	1,328	109,831	34,521 (31.43)	2.27 (2.27, 2.27)
	2023	1,334	99,531	25,360 (25.48)	2.25 (2.25, 2.25)
**Guinea pig**	2014	395	9,181	3,423 (37.28)	0.91 (0.91, 0.91)
	2015	499	12,593	5,311 (42.17)	0.90 (0.90, 0.90)
	2016	654	14,653	5,908 (40.32)	0.92 (0.92, 0.92)
	2017	848	16,907	6,125 (36.23)	0.94 (0.94, 0.94)
	2018	955	18,283	6,489 (35.49)	0.95 (0.95, 0.96)
	2019	1,051	25,322	9,758 (38.54)	0.96 (0.96, 0.96)
	2020	1,052	23,051	7,359 (31.92)	0.97 (0.97, 0.97)
	2021	1,114	26,000	7,922 (30.47)	0.98 (0.98, 0.98)
	2022	1,244	24,649	6,935 (28.14)	0.99 (0.99, 0.99)
	2023	1,259	23,329	6,149 (26.36)	0.97 (0.97, 0.97)

Juvenile age up to 14 months (dog), 13 months (other species).

When comparing to alternative methods of calculating mean population bodyweight, the period mean bodyweight of the whole population as recorded in clinical records substantially underestimated the model-adjusted point mean population bodyweight for all species, while excluding animals under two years of age as done in previous surveys [1] resulted in overestimation of point mean whole population bodyweight (Fig 2).

## Discussion

Accurate estimation of point mean population-level bodyweight for different species is vital to ensure confidence in calculations based on this measure. The methodology for estimating this figure in the current study ensures that all patient ages are represented in a way that reflects the age distribution within the species population, and by adjusting for juvenile growth over time, allows bodyweight measurements recorded at disparate times to be combined to estimate population point mean bodyweight on a specific target date. Now developed, the models presented here are straightforward to reuse to calculate point mean population bodyweights with data collected from patients in the future.

One major application requiring point mean population bodyweight is calculation of the DDDVet, which aims to provide “standardised units of measurement for the reporting of data on antimicrobial consumption by species” [39]. By taking into account differences in recommended dosages between different drugs and species, this measure improves standardisation when reporting data on antimicrobial usage. Application in national benchmarking and comparison between countries can support progressive improvements in antimicrobial stewardship [40]. Calculation of DDDVet per animal in small companion animal species currently relies on assumed standard bodyweights of 20 kg for dogs and 5 kg for cats following European Commission (EC) guidelines [40], or 18.60 kg and 4.43 kg respectively following UK guidelines (D. Singleton and Veterinary Medicines Directorate, UK, written communication, 2021). However, as these UK standard bodyweights are calculated from adult animals only, and the EC guidelines do not describe the method of calculation [40], the skewing effect on mean population bodyweight of less heavy juvenile animals does not appear to have been included in previous estimates.

Inclusion of the full age range of small companion animal patients in point mean species bodyweight is important for calculations such as DDDVet per animal, where drugs of interest, including antimicrobials, are prescribed to patients of all ages [41]. As DDDVet per animal is inversely proportional to the total population bodyweight at risk (S1 Formula) [1] and period mean population bodyweight calculated from adult animal bodyweights only is higher than point mean bodyweight that includes juveniles, this risks substantial underestimation of antimicrobial use when calculated as DDDVet per animal per unit time. By using values for point mean whole-population bodyweights that account for juvenile growth, a more accurate estimation of DDDVet per animal can be obtained.

In humans the DDD is ‘…the assumed average maintenance dose per day for a drug used for its main indication in adults’, and is based on the assumption that the bodyweight of a ‘standard’ human adult is 70 kg [9]. The DDD for drugs used in children is calculated using adult bodyweight assumptions in most cases, and so the skewing effect of juvenile bodyweight is not taken into account when calculating DDD in people. As noted earlier, annual prevalence rates of use of antimicrobials in children (≤14 years of age) appears to be similar overall to that of adults ( ≤ 65 years) [10], suggesting that increased consideration of juvenile bodyweight profiles may improve accuracy of DDD calculation in human patients.

The generalisability of veterinary practice derived standard bodyweights in this study is likely to depend on the intended application. It has been estimated that only 77% of dogs in the UK are registered with veterinary practices [42], and certain breeds and age groups may be more likely to present for veterinary care than others [12,43]. Thus, it is unclear to what extent bodyweights derived from veterinary patients are representative of the wider UK companion animal population that does not have access to veterinary care. Based on population estimates from 2023 [44], this study derived bodyweights from 19.98% of all owned dogs, 10.54% of all owned cats, and 9.05% of all owned rabbits in the UK. Therefore, the standard bodyweights presented here are very likely to be valid for applications concerning the population under UK veterinary care, such as calculation of DDDVet per animal, insurance premiums, or monitoring of health trends in veterinary practice. However, further work to estimate the effect of potential selection bias may be valuable to clarify their validity for the wider population, for example in estimating the environmental impact of companion animal nutrition [45], or monitoring of health trends in unowned animals.

The 13-month duration of juvenile growth in rabbits and guinea pigs identified in the current study was substantially longer than growth periods of laboratory populations reported in previous studies. Cessation of juvenile growth in rabbits has previously been reported between nine to ten months of age [46], and around eight months in guinea pigs [47]. These laboratory studies did not include animals over one year of age and animals may have differed in genetic and nutritional status compared to the privately-owned companion animals included in the current study. This suggests that growth profiles reported in laboratory studies may not always be appropriate for small mammals of the same species when kept as pets.

Mean bodyweights of the populations of cats, rabbits, and guinea pigs under primary veterinary care in the UK appear to have remained similar between 2013 and 2023. However, for dogs there was a trend towards lower bodyweight, perhaps reflecting changing owner preferences for smaller breeds over time [48]. This highlights how historical selective breeding of dogs has resulted in far more phenotypic variation than in other species where selection of extreme size conformations is lower [13], and suggests that annual updating of point mean species bodyweight in dogs would be beneficial to maintain accuracy in DDDVet calculations in future years.

## Limitations

While this study demonstrated excellent model fit as indicated by the observed calibration statistics, it is accepted that there remain some limitations to the accuracy of DDDVet calculation using the standard bodyweights presented here. The current methodology does not consider potential variation in the frequency of prescribing between different juvenile age bands, meaning that in smaller samples, calculated usage may be confounded by variation in juvenile age distribution and age-related prevalence of infectious diseases between different practices. Similarly, variation between breeds is not specifically addressed. The standard bodyweights presented here are intended for population-level measures, and were calculated using sample sizes sufficiently large that the distribution of patient characteristics such as sex, age, and breed was very likely to be representative of the wider population. Patient characteristics in smaller samples may diverge from the broader population due to individual variation, and as these characteristics can impact disease incidence and the use of different medications [41,49], population-level standard bodyweights may have limited validity when sample size is small.

Further work investigating potential variation in drug utilisation rates between different breeds and juvenile age bands may be helpful to further improve accuracy of DDDVet calculations at a smaller subpopulation or practice level.

## Conclusions

Estimated values for point mean species bodyweights were substantially lower than those used currently for calculation of national benchmarks that report DDDVet and assume a standard bodyweight of 18.60 to 20.00 kg for dogs, and 4.43 to 5.00 kg for cats [1]. Use of point-mean species bodyweights adjusted for juvenile growth is likely to reduce bias in measures where the population at risk includes patients of all ages. Variation in annual point-mean bodyweight for dogs suggests that annual updates of standard canine bodyweight values are needed. As international generalisability would require similar breed and age structures between other countries and the UK for the animals under veterinary care, and also similar bodyweights between animals of the same breed, varying patterns of dog breed ownership internationally may necessitate country-specific standard bodyweight values for this species.

## Supporting information

S1 Formula Formula for the daily defined dose for veterinary species (DDDVet) per animal. (DOCX)

S2 Formula Form of prediction model to calculate proportional bodyweight change in juvenile animals. (DOCX)

## Abbreviations

DDD: daily defined dose (humans)
DDDVet: defined daily dose for veterinary species
EMA: European Medicines Agency
WHO: World Health Organisation
