# Supplementary material for: Calculation of standard bodyweights for dogs, cats, rabbits, and guinea pigs
Source: PLoS One. 2025 Feb 13;20(2):e0318734. doi: 10.1371/journal.pone.0318734 (PMC11825090; doi:10.1371/journal.pone.0318734)
Supplement: S1 Formula — (DOCX) [file pone.0318734.s001.docx]

**S1 Formula**. Formula for the Daily Defined Dose for veterinary species (DDDVet) per animal.

$$DDDVet/animal = \frac{Total amount of active ingredient (mg)}{DDDVet (mg/kg/day) * total animal population weight at risk (kg)}$$

Where: ‘DDDVet’ represents a recommended 24-hour dose of the active ingredient

‘Total animal population weight at risk’ is calculated by multiplying standard

species bodyweight by the number of animals of that species in the population

at risk*.*

Veterinary Medicines Directorate. 2022. Veterinary Antibiotic Resistance and Sales Surveillance Report (UK-VARSS 2021). Available: <https://www.gov.uk/government/publications/veterinary-antimicrobial-resistance-and-sales-surveillance-2022>
