# Supplementary material for: Calculation of standard bodyweights for dogs, cats, rabbits, and guinea pigs
Source: PLoS One. 2025 Feb 13;20(2):e0318734. doi: 10.1371/journal.pone.0318734 (PMC11825090; doi:10.1371/journal.pone.0318734)
Supplement: S2 Formula — (DOCX) [file pone.0318734.s002.docx]

**S2 Formula**. Form of prediction model to calculate proportional bodyweight change in juvenile animals.

$$y_{i}=\beta_{0}+\beta_{1}Age_{i}+\beta_{2}Age_{i}^{2}+\beta_{3}Age_{i}^{3}+\beta_{4}Interval_{i}+\beta_{5}Interval_{i}^{2}+\beta_{6}Interval_{i}^{3}+\beta_{7}(Age_{i}\times Interval_{i})$$

Where: y­*_i_* is the proportional change in bodyweight (kg) for animal *i.*

Age is the age in months of animal *i* at the time of calculation*.*

Interval is the time in months between the date of bodyweight measurement and

the date of calculation.
